# Electrospun Nanomaterials: Applications in Food, Environmental Remediation, and Bioengineering

**DOI:** 10.3390/nano10091714

**Published:** 2020-08-29

**Authors:** Alberto Falco, Ricardo Mallavia

**Affiliations:** Institute of Research, Development and Innovation in Biotechnology of Elche (IDiBE), Miguel Hernández University (UMH), 03202 Elche, Spain

Among the large number of methods to fabricate nanofibers, electrospinning stands out because of its simplicity and versatility. The formation of nanoscaled fibers via electrospinning is based on the application of high voltage (usually ranging from 1 to 30 kV) to generate an electrostatic field that induces the formation and stretching of a jet from a viscoelastic polymer solution or melt. The nanofibers are finally formed by either evaporation of solvent or freezing of the melt. Regarding the setup, one of the electrodes can be placed either directly in this solution, or onto the metal needle attached to the tip of the syringe feeding the solution at a constant and controllable flux by means of an infusion pump. The other electrode is connected to a metal object that can work as collector (that can be covered by a fabric), usually a static plane surface that is located perpendicular and at a certain distance from the spinneret. As a result of the forces involved, a highly electrified continuous jet is ejected from the pendent drop of solution at the top of the spinneret and deposited on the collector as randomly distributed nanofibers. In addition, by modifying the basic setup of electrospinning and/or the composition of the electrospinnable solution, the morphology (including porosity), diameter and functionality of the final outcome can be controlled. For instance, nanofibers can even be aligned by adapting the collector to a rotary cylinder or disposed in a core/shell structure by using a spinneret with two coaxial capillaries supplying two solutions separately [1,2,3,4].

The origin of this method, which allows the efficient obtention of long, uniform nanofibers with either solid or hollow interiors, dates back to the beginning of the 20th century, when some essential technical milestones for its development, such as the generation and manipulation of electricity, were reached. However, a series of other preceding scientific advances paved the way towards this invention, which can be considered as a variant of the electrospraying process (i.e., the collapse of liquid jets into droplets by the effect) [3,4,5]. Among them, the distortion and attraction of liquid droplets when applying electrostatic forces, reported by William Gilbert in 1600, could be considered as the oldest one. In the middle of the 18th century, George Mathias Bose described the generation of aerosols by the application of high electric potentials to fluids, and Giovanni Battista Beccaria observed that when fluids were charged, they evaporated faster. Such discoveries might be considered as the basis for the development of electrospraying. It was not until the verge of 19th century that John William Strutt (Lord Rayleigh) first observed the electrospinning phenomena, and Charles Vernon Boys first designed and constructed an electrospinning device and drew fibers from a number of melts, mostly molten waxes. It was in 1900 and 1902 when John Francis Cooley and William James Morton, respectively, filed the first electrospinning patents on industrial applications, and a bit later when John Zeleny studied in detail the mechanisms underlying the process (mostly electrospraying). The origin of electrospinning was established with broad consensus in 1934, when Anton Formhals started patenting several inventions on the technology associated to this process. After up to 22 patents in about 10 years, Formhals greatly improved the process and made electrospinning an efficient and viable technique.

Later, the work of Sir Geoffrey Ingram Taylor in the 1960s, whose fundamental studies on the jet forming process laid the theory groundwork for electrospinning, is of note. Since then, the conical shape of the jet occurring as a consequence of the distortion of the spinneret droplet when the electrostatic forces exceed its surface tension has been referenced as the “Taylor cone” in later literature [6]. More recently, Larrondo and Manley in the early 1980s, and the Reneker’s group in the early 1990s, notably revitalized this technology by demonstrating the possibility of electrospinning a range of molten polymers [7] and organic polymer solutions [8,9], respectively. Reneker also popularized the term “electrospinning”, which derives from the former “electrostatic spinning” used until then. In the last decades, the advances in the fabrication, processing, and characterization of electrospun nanofibers have contributed to the wide expansion of this technique across laboratories and industry. This growth is mainly promoted by the surging interest in nanotechnology and the great expectations placed on the unique properties of nanomaterials, with notable support from the outstanding progress of the materials and polymer sciences in recent times [4].

As for the raw materials used for electrospun nanofibers, polymers comprise an unlimited number of molecules with different properties that can even be endowed with extra specific features by means of feasible functionalization protocols. In addition, electrospun nanofibers can be prepared from not only single/pure polymer sources, but also compatible polymer blends to combine the properties of their moieties [10]. Altogether, this family of compounds guarantee an extraordinary diversity of nanofiber compositions and thus properties, which explains the broad application potential of these nanomaterials. Indeed, depending on their specific composition/properties, electrospun nanofibers can be exploited in multiple applications covering areas as different as nanoelectronics, energy storage, catalyst substrates, sensors, nanofilters, protective and smart clothing, and adsorbent and biomedical materials [11,12,13,14,15].

At this point, and regardless of the application, it is worth mentioning that the assessment of the environmental impact of the nanomaterials used, as well as their fabrication and degradation by-products, is critical to avoid possible harmful effects on ecosystems by allowing, for instance, the design of appropriate disposal protocols for these compounds and to preferentially opt for those that are eco-friendly. In this sense, polymers also offer a large collection of both natural, but also synthetic, electrospinnable compounds that are non-toxic and biodegradable, as well as biocompatible [4,10]. Electrospun nanofibers made of such biomaterials are thus suitable for applications involving direct (and indirect) contact with biological systems, which mostly comprise applications within the biomedical [1,4,11,13,16,17,18,19], but also the environmental protection [11,16] and the food packaging fields [11,20,21].

The present book compiles the Special Issue “Electrospun Nanomaterials: Applications in Food, Environmental Remediation, and Bioengineering” from the journal “Nanomaterials”, and, therefore, it comprises several review and research articles addressing several applications of electrospun nanofibers in these areas. In regard to the application of these nanomaterials to the food field, the implementation of electrospinning in food packaging is thoroughly revised in Zhao et al. (2020) [22], which also includes a summary of the additional characteristics provided by functional food packaging materials, degradability, superhydrophobicity, edibility, antibacterial activity and high barrier protection, as well as the contribution of electrospun nanofibers to their development. In terms of environmental remediation, this topic is tackled by two research articles that converge on the green/sustainable generation of energy by improving two different applications (i.e., microbial fuel cells [23] and solar thermal techniques [24]) using electrospun nanofibers.

The current research and utilization of nanofibers mainly for biomedical applications is proportionally covered in this compilation. In this sense, the biomedical applications of electrospun nanofibers included here can be classified into two broad types: drug delivery systems and tissue scaffolds. Regarding drug delivery, polymers comprise a large number of biocompatible materials with an extraordinary versatility to be structured as different nanomaterials with drug-loading capacity. Thus, compounds with different solubility properties can be encapsulated into polymeric nanomaterials by either changing the polymer source or the nanomaterial type. Here, this is shown by Mira et al. (2020) [25] for the encapsulation of different classes of antibiotics by using two separate derivatives of poly(methyl vinyl ether-alt-maleic anhydride) (PMVE/MA) that can be used (alone or in combination with other polymers such as fluorescent polyfluorenes [26,27,28]) for the fabrication of both nanoparticles [29] and electrospun nanofibers [30,31]. Polymeric nanofibers also protect loaded compounds from degradation, as described by Cruz-Salas et al. (2019) [32] for electrospun nanofibers made from agave fructans, which thermoprotect and photoprotect encapsulated β-carotene. Another advantage of polymeric nanofibers is their modifiable drug-release kinetics by means of feasible design changes to adjust their degradability or porosity for providing optimal therapeutic drug concentrations. As reported here [33,34], this property is being intensively investigated at present for the development of improved dressings, bandages or coatings with, for example, antibacterial activity. In this sense, the use of functional polymers such as chitosan (with reported protective immunomodulatory properties) is also attracting great interest, as widely reviewed by Maevskaia et al. (2020) [35].

Finally, the current great effort made by the scientific community in the development of tissue scaffolds based on electrospun nanofibers is also addressed here. The work of Miroshnichenko et al. (2019) [36] provides a representative example of the research lines in this area by reporting the cell interaction improvements when coating polycaprolactone nanofibers with covalently bonded platelet-rich plasma. Likewise, Li et al. (2019) [37] broadly review the progress in the particular area of electrospun polyvinylidene fluoride-based materials used for bone and neural tissue engineering.

In summary, the papers collected in this Special Issue entitled “Electrospun Nanomaterials: Applications in Food, Environmental Remediation, and Bioengineering” illustrate the high diversity and potential for implementation of electrospun nanofibers in these fields, including the covering of a wide number of subtopics. Undoubtably, such pieces of fundamental research will contribute to the promotion of electrospinning as the focal point in the future development of technological applications at the interface of biological systems, which promise long-term benefits for both health and the environment.
